# The Association Between Multilingual Experience Factors and Cognitive Functioning in Older Adults: A Lifelines Study

**DOI:** 10.1093/geronb/gbae200

**Published:** 2025-01-11

**Authors:** Floor van den Berg, Jelle Brouwer, Hanneke Loerts, Remco Knooihuizen, Merel Keijzer,

**Affiliations:** Linguistics and English as a Second Language, Faculty of Arts, University of Groningen, Groningen, the Netherlands; Linguistics and English as a Second Language, Faculty of Arts, University of Groningen, Groningen, the Netherlands; Applied Linguistics, Faculty of Arts, University of Groningen, Groningen, the Netherlands; Linguistics and English as a Second Language, Faculty of Arts, University of Groningen, Groningen, the Netherlands; Linguistics and English as a Second Language, Faculty of Arts, University of Groningen, Groningen, the Netherlands; (Psychological Sciences Section)

**Keywords:** Cognition, Multilingualism, Population-based cohort study

## Abstract

**Objectives:**

The complex life experience of speaking two or more languages has been suggested to preserve cognition in older adulthood. This study aimed to investigate this further by examining the relationship between multilingual experience variables and cognitive functioning in a large cohort of older adults in the diversely multilingual north of the Netherlands.

**Method:**

A total of 11,332 older individuals participating in the Lifelines Cohort Study completed a language experience questionnaire. From this cohort, a subset was selected (*n* = 3,972, aged 59–86) for whom complete demographic and cognitive data were available and who had learned at least two languages to evaluate the association between multilingual experience variables and cognitive functioning. Cognitive functioning was assessed using the Cogstate Brief Battery, which measures processing speed, attention, working memory, and recognition memory.

**Results:**

A linear regression analysis revealed that a higher number of languages learned was related to better performance on all subtasks. In addition, a later onset of acquisition of the second language (L2) was associated with better attention. These effects were independent of demographic variables such as age, education level, income level, and country of birth.

**Discussion:**

The results demonstrate that in our cohort only the experience factors of the number of languages learned and L2 onset of acquisition related to cognitive functioning. Our evidence supports the idea that there is a positive relationship between multilingual experiences and cognitive functioning in older adulthood, but more longitudinal work is needed to establish whether learning multiple languages can potentially promote healthy aging.

Cognitive functions are known to decline with age due to the loss of gray matter, white matter, and functional connectivity in the brain (Craik and Salthouse, 2011). However, some older adults seem to be more resilient to the cognitive consequences of brain atrophy and demonstrate cognitive decline to a lesser extent than their peers: they show higher levels of so-called cognitive reserve. Cognitive reserve has not only been proposed to attenuate typical cognitive aging but also to act as a compensatory mechanism for pathological brain atrophy associated with dementia (Stern, 2012): a higher cognitive reserve has been linked to a delayed onset of mild cognitive impairment (MCI) or Alzheimer’s disease (AD) symptoms (Soldan et al., 2020), a lower diagnostic conversion risk to AD, and slower cognitive decline in predementia stages (Van Loenhoud et al., 2019), thereby contributing to more cognitively healthy years. As such, building up cognitive reserve could be a promising strategy to achieve healthier aging in our rapidly aging world population.

Several life experiences and lifestyle factors have been suggested to augment cognitive reserve, including but not limited to a higher level of education (Meng and D’Arcy, 2012), physical activity (Blondell et al., 2014), and engagement in (complex) activities such as playing a musical instrument (Amer et al., 2013). Bilingualism, being a complex and often sustained life experience (Bialystok, 2017), has also been proposed as a contributor to cognitive reserve (Bialystok, 2021). However, it remains unclear which specific elements of bilingual experience contribute to this protective effect (Voits et al., 2022). For example, is it the mere knowledge of multiple languages, their usage, or a combination of both that supports cognitive performance in later life? In addition, multilingualism, often defined as the use of three or more languages, has been suggested to offer greater effects on cognitive aging than bilingualism in some circumstances (Schroeder and Marian, 2017), but this literature is still limited in comparison to the work on the cognitive effects of bilingualism. Understanding whether, and how, these aspects of language experience are associated with cognition in older adulthood could further clarify the role of multilingual experience in cognitive reserve. This study therefore set out to not only illuminate the potential additive effect of knowing more than two languages but also specific second-language variables to better understand their relationship with cognitive functioning in older adulthood.

Psycholinguistic research has consistently found that the languages in a bilingual mind are always simultaneously activated (Bialystok et al., 2012). This constant co-activation of languages is argued to be regulated through language control, which overlaps to some degree with domain-general cognitive control processes (Kroll and Bialystok, 2013) and has been suggested to have positive consequences for the mind and brain. Acquiring and speaking two languages has in fact been shown to dynamically alter brain structure and function over time (Pliatsikas, 2020), effectively increasing and maintaining the efficiency of cognitive functions such as processing speed (Grant and Dennis, 2017), executive functioning (Ware et al., 2020), working memory (Grundy and Timmer, 2017), and memory recall (Schroeder and Marian, 2014) across the lifespan.

It has to be pointed out that bilingualism has not been consistently found to result in cognitive adaptations (Leivada et al., 2021). Even in the presence of neural consequences of bilingual experience, behavioral cognitive effects cannot always be detected (DeLuca et al., 2020), especially in young adult populations who tend to perform optimally on a cognitive level (Bialystok et al., 2012). Importantly, the cognitive effects of bilingualism are more consistently found in older adults, in whom individual differences in cognitive abilities are most pronounced due to differences in accumulated cognitive reserve (Bak, 2016). These cognitive adaptations associated with bilingualism also seem to have clinical implications: actively speaking two languages for a lifetime has been found to delay the onset of MCI (Calabria et al., 2020) and AD symptoms (Anderson et al., 2020). In sum, lifelong bilingualism may be a promising way to build up cognitive reserve, and thus maintain better cognitive health in older adulthood.

Traditionally, the association between bilingualism and cognitive reserve has been investigated by comparing bilinguals to their monolingual peers. These comparisons laid the foundation for current work and have yielded encouraging results (discussed earlier). However, previous studies have often treated bilinguals as a homogeneous group in their design, which masks the inherent complexity and variability of bilingual experiences (Leivada et al., 2021). Indeed, the field is currently moving away from treating bilingualism as a one-size-fits-all concept: recent papers have advocated capturing the complexity of bilingual experiences using more sensitive, non-dichotomous measures (Anderson et al., 2018; DeLuca et al., 2019; Gullifer and Titone, 2020). These experiences comprise knowledge-based factors, which are largely static (e.g., language proficiency level and age of onset of acquisition [AoA] of these languages) as well as usage-based factors, which are more dynamic (e.g., language use patterns and the degree of switching between languages). Especially variations in language use have been argued to place different demands on cognitive functioning, necessarily resulting in different cognitive adaptations in individuals (Green and Abutalebi, 2013).

Perhaps an even more complex life experience than bilingualism is speaking more than two languages, which could therefore offer relatively greater benefits in cognitive aging. Several studies have found an additive effect of multilingualism compared with bilingualism in terms of AD or MCI onset (as reviewed in Schroeder and Marian, 2017). Furthermore, in two large-scale studies, it was found that a higher number of languages spoken was related to better verbal abilities and processing speed (Ihle et al., 2016), and to a better “cognitive state” (Kavé et al., 2008). Strikingly, this additive effect of speaking multiple languages was found independently of demographic variables (e.g., age, immigration status, and years of education). Still, despite the fact that a considerable part of the world population is multilingual, multilingual populations are currently critically underrepresented in the literature on language experience and cognition more generally.

The goal of the present study is to examine the relationship between cognitive functioning and both knowledge-based and usage-based language experience factors in a large cohort of cognitively healthy older adults (55+) in the north of the Netherlands with varying multilingual experiences (see Author Note 1). Although 85.2% of respondents in the Netherlands do not regularly use another language in addition to Dutch (Taalunie, 2019), and can be characterized as functionally monolingual, the Netherlands is overall often regarded as a highly multilingual setting. Indeed, most will have been taught foreign languages in secondary education (e.g., English, German, and French). In addition, a vast majority have at least some proficiency in English due to exposure to English through media consumption (Edwards, 2016). Furthermore, many people in the northern Netherlands speak a regional language in addition to Dutch. This may be particularly true for the older demographic; surveys on regional language use in the Netherlands have found that 39.8% of the respondents older than 60 living in provinces in the north-east (i.e., Overijssel, Gelderland, Groningen, and Drenthe), speaks Low Saxon (Bloemhoff, 2005), and 69% of inhabitants of Fryslân older than 65 speaks Frisian well or very well (Klinkenberg et al., 2018). In sum, the north of the Netherlands serves as a unique, diversely multilingual landscape for the present study in which to study the association between multilingual experience and cognition in older adulthood.

To the best of our knowledge, only two studies so far have investigated the cognitive effects of multilingual experience in this particular population. Pot et al. (2018) examined the contribution of both knowledge-based and usage-based multilingual experience variables alongside other possibly modulating factors involved in the cognitive aging process in a sample of 387 healthy individuals aged 65 years or older. Enhanced cognitive control was found for those participants who were open to new experiences and predominantly used their second language (L2) across social domains, but the knowledge-based variables (e.g., number of languages spoken and onset of bilingualism) did not relate to cognitive control. Nijmeijer et al. (2022) found, in an even larger sample of Dutch older adults in the northern Netherlands (the same sample that forms the basis of the present study; see later), that having a multilingual experience, in particular when combined with a musical experience, was associated with better cognitive performance in comparison to having no to limited musical or multilingual experience. Although participants were classified according to both knowledge-based and usage-based multilingual experience variables (the number of languages learned, current multilingual language use, and frequency of multilingual language use), how these variables related to cognitive functioning was not assessed.

The present study adds to this work by exploring the association between multilingual experience and cognitive functioning in a large cohort of older adults using a comprehensive language experience profile, comprising knowledge-based and usage-based multilingual experience variables. We do so on the basis of the Lifelines Cohort Study, which employs the Cogstate Brief Battery to measure processing speed, attention, working memory, and recognition memory (see Methods). Considering the evidence that speaking two languages offers better performance on processing speed (Grant and Dennis, 2017), working memory (Grundy and Timmer, 2017), and memory recall (Schroeder and Marian, 2014) when compared with monolingualism, and that speaking a higher number of languages may have an additive effect on cognitive functioning (Ihle et al., 2016; Kavé et al., 2008), it is expected that having learned more languages in life will be positively associated with performance on these cognitive tests. In addition, we explore how individual differences in multilingual experience related to the L2 and language use patterns may be associated with this performance. The current study, with its unprecedentedly large sample size, can uniquely shed more light on the relative relationships between knowledge-based versus usage-based multilingualism variables and cognition in later life.

## Method

### Study Context

This study used data from Lifelines, a multidisciplinary, prospective, population-based cohort study tracking the health and health-related behaviors of almost 168,000 participants (approximately 10% of the population) from three generations in the north of the Netherlands (see Scholtens et al., 2015). Specifically, the Lifelines study employs a broad range of investigative procedures in assessing the biomedical, socio-demographic, behavioral, physical, and psychological factors that contribute to the health and disease of the general population, with a special focus on multimorbidity and complex genetics. Participants were recruited in waves between 2006 and 2013, with the first wave being contacted through general practitioners and the rest of the participants through already-included participants and online self-registration. The Lifelines study adheres to the principles of the Declaration of Helsinki and is in accordance with the research code of the University Medical Center Groningen (UMCG), the Netherlands. The Lifelines study was approved by the medical ethical committee of the UMCG (ref. no. 2007/152). All participants provided written informed consent.

### Language Experience Questionnaire

A digital language experience questionnaire was distributed through the Lifelines infrastructure in December 2019 to approximately 20,000 participants aged 55 years or older in the second Lifelines assessment period (between 2014 and 2018). The questionnaire, based on Pot et al. (2018), was completed in Dutch by 11,332 participants (response rate 57.6%) between December 2019 and April 2020. A version translated into English is available in the Supplementary Material. Several variables related to the knowledge and use of multiple languages were extracted from this questionnaire and used in the analysis. These were the knowledge-based variables of number of languages learned (including regional languages), self-rated L2 speaking proficiency on a scale of 1-10, and age of onset of L2 acquisition (L2 AoA), and the usage-based variables of frequency of using multiple languages (ordinal), and frequency of language switching (categorical). Participants responded to the questions for proficiency and AoA for up to six languages and could indicate if they spoke more than six languages.

### Cognitive Measures

For the purpose of this study we used outcomes from the Cogstate Brief Battery as a measure of multiple cognitive domains (Kuiper et al., 2017). The tasks in the Cogstate Brief Battery have been demonstrated to have strong construct validity, high test–retest reliability, and high sensitivity to changes in cognitive function (Fredrickson et al., 2010; Maruff et al., 2009). This computerized battery was administered on-site, with instructions in Dutch, during the second Lifelines assessment period (2014–2018). This assessment did not include other measures of cognitive functioning. The Cogstate Brief Battery measures four cognitive domains on the basis of four playing card tasks, in the following fixed order:

1. Detection task (processing speed)
2. Identification task (attention)
3. One Back task (working memory)
4. One Card Learning task (recognition memory)

A detailed description of the tasks is provided in the Supplementary Material. Participants completed the tasks on a computer, using a keyboard. The primary outcome measures were reaction times (RTs) for the Detection and Identification tasks and the proportion of correct answers (hit rate) for the One Back and the One Card Learning tasks. Hit rates and RTs were automatically transformed by the Cogstate software into normalized distributions using arcsine square root and log10 transformation, respectively.

### Statistical Analysis

**Figure 1.**
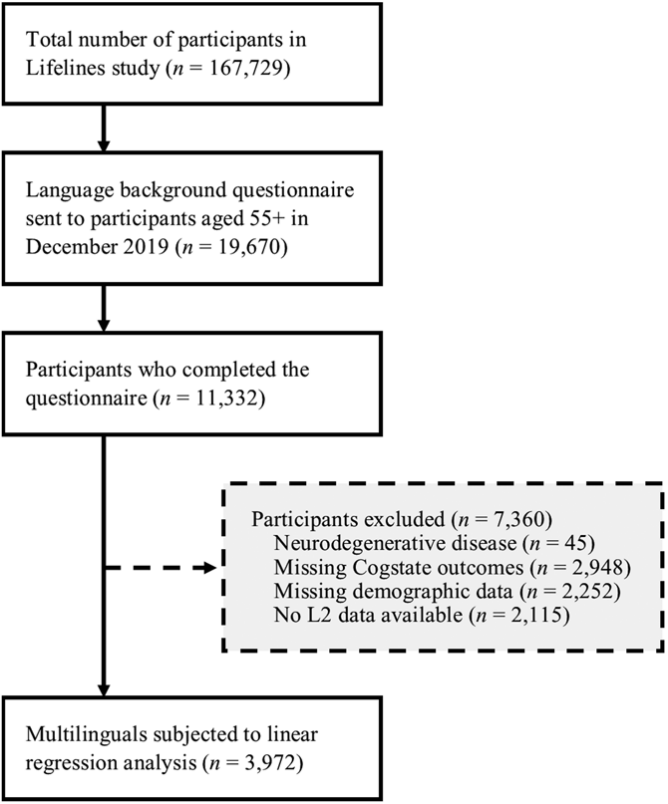
Flowchart of the final sample selection procedure.

The data preparation and model building procedure are described in detail in the Supplementary Material. Subjects diagnosed with a neurodegenerative disorder known to affect cognitive functioning, including dementia, Parkinson’s disease, and multiple sclerosis, were excluded from the analysis (*n* = 45). Participants for whom no Cogstate outcomes were available (*n* = 2,948) or had missing socioeconomic data (*n* = 2,252) were also excluded, leaving 6,038 participants. From this sample, we derived a multilingual group (*n* = 3,972, 1,942 women, *M*_*age*_ = 65.93, *SD*_*age*_ = 4.37, age range: 59–86) by selecting the participants for whom questionnaire data were available for two or more languages. The final sample selection procedure is summarized in Figure 1.

The primary outcomes from each Cogstate subtest were subjected to linear regression analysis in R (version 4.2.0, R Core Team, 2022) and were entered as dependent numerical variables. We used stepwise modeling with the Akaike Information Criterion (AIC) to evaluate the predictive value of multilingual experience variables for cognitive functioning, retaining only predictors that significantly improved the model fit. As a starting point, a demographics model was built to account for potential confounding effects, including the following covariates: age at assessment, sex, educational attainment, net family income level as a proxy of socioeconomic status, and country of birth as a proxy of immigration status. This was done because such factors have been associated with cognition and cognitive aging more generally (Farah et al., 2006; Fuller-Thomson and Kuh, 2014; Nooyens et al., 2022). Educational level in particular can be a confound in the relationship between multilingual experience and cognition, considering that participants with higher educational attainment levels may have learned additional languages at school. Educational attainment was categorized as low (elementary education), middle (secondary education), or high (tertiary education). Family income level was categorized as lower (<2,000 euros/month), middle (2,000–3,000 euros/month), or higher (>3,000 euros/month). Country of birth was dichotomized into “the Netherlands” and “other country.”

## Results

### Sample Characteristics

Descriptive statistics on demographic and multilingual experience variables of the sample are given in Table 1. As shown in this table, the number of languages learned in this sample ranged from two to seven or more: 11% indicated that they had learned two languages, and another 15% that they had learned three languages. The majority had either learned four (22%) or five (35%) languages. Another 12% had learned six languages and a smaller portion indicated to have learned seven or more languages (5%). Note that the questionnaire prompted participants to name the languages *learned*, and this may include languages they do not *use* frequently (anymore).

**Table 1.** Descriptive Statistics of Sample Demographics and Multilingual Experience.

Variable	Item	*M* (*SD*)	min–max	*n* (%)
*Demographics*				
Age at assessment		65.93 (4.37)	59–86	
Age at questionnaire		70.82 (4.26)	65–91	
Sex	Men			2,030 (51%)
	Women			1,942 (49%)
Educational attainment ^a^	Low			1,405 (35%)
	Middle			1,042 (26%)
	High			1,525 (38%)
Income level ^b^	Low			1,021 (26%)
	Middle			1,537 (39%)
	High			1,414 (36%)
Country of birth	The Netherlands			3,899 (98%)
	Other country			73 (2%)
*Language experience*				
Self-reported multilingualism	Yes			3,106 (78%)
	No			866 (22%)
Number of languages learned		4.39 (1.33)	2–7 or more	
L2 speaking proficiency		8.25 (1.76)	1–10	
L2 age of onset of acquisition		7.49 (6.22)	0–71	
Frequency using multiple languages	Every day			1,348 (34%)
	More than once a week			1,120 (28%)
	Once a week			490 (12%)
	Once a month			381 (10%)
	Less than once a month			633 (16%)
Language switching habits	No			2,068 (52%)
	Sometimes			1,610 (41%)
	Frequently			294 (7%)

*Notes*: SD = standard deviation. Sample of *n* = 3,972. Percentages are rounded.

^a^“Low”: no education, primary education, lower or preparatory secondary vocational education, and junior general secondary education. “Middle”: secondary vocational education or work-based learning pathway, senior general secondary education, and pre-university secondary education. “High”: higher vocational education and university education.

^b^“Low”: <2,000 euros/month. “Middle”: 2,000–3,000 euros/month. “High”: >3,000 euros/month.

A variety of learned languages was reported by the participants (see Supplementary Table 1). Almost 55% of the respondents reported Dutch as their first language (L1), and approximately 23% and 19% of the participants spoke Frisian or Low Saxon as their L1, respectively. Almost 41% spoke Dutch as their L2 (presumably constituting many of the L1 Frisian and L1 Low Saxon speakers); almost 24% indicated that they spoke English as their L2, and approximately 8% and 13% of the respondents spoke Frisian and Low Saxon as their L2, respectively. On average, participants self-rated their L2 speaking proficiency with an 8.25 out of 10 and started to learn their L2 at 7.49 years of age.

Regarding the frequency of multilingual language use, 34% of participants indicated using multiple languages on a daily basis, followed by more than once a week (28%), once a week (12%), less than once a month (16%), and once a month (10%). Language switching was not a universal characteristic of our sample, as the majority (52%) indicated not switching between their languages and only using a single language per context or setting; 41% indicated sometimes switching between their languages within a context or setting, and only 7% reported to frequently switch. Despite having learned and used several languages to some extent, not all participants self-categorized as multilingual (78% did). Additional descriptive statistics of proficiency, age of onset of acquisition, and learning context of all the languages learned by our sample are reported in Supplementary Table 2.

### Cogstate Performance and Multilingual Experiences

Descriptive statistics of the raw outcomes for each Cogstate subtask can be found in Table 2. Statistics of the final regression models, showing the relationships between multilingual experience factors and processing speed (Detection task), attention (Identification task), working memory (One Back task), and recognition memory (One Card Learning task) are summarized in Table 3. Spearman’s rho correlations between the demographic and multilingual experience variables included in the analyses are listed in Supplementary Table 3. Effect sizes for each predictor in the models are provided in Table 4.

**Table 2.** Descriptive Statistics of the Raw Primary Outcomes for Each Cogstate Subtask (*n* = 3,972 participants)

Outcomes	*M* (*SD*)
Detection task (reaction time in ms)	520 (319)
Identification task (reaction time in ms)	560 (146)
One Back task (hit rate)	0.87 (0.15)
One Card Learning task (hit rate)	0.64 (0.11)

*Note*: SD = standard deviation.

**Table 3.** Linear Regression Model Summaries for the Final Models Including Significant Multilingual Experience Predictors

Variable	Est.	SE	*t*	*p*	95% CI	Adjusted *R*^2^
DET (*n* = 3,864)						0.063
Effect (intercept)	2.672	0.006	424.20	**<.001** ***	[2.659, 2.684]	
Number of languages	−0.017	0.003	−5.71	**<.001** ***	[−0.022, −0.011]	
Age	0.035	0.003	13.06	**<.001** ***	[0.030, 0.040]	
Sex (−F + M)	−0.012	0.006	−2.17	**.030** *	[−0.023, 0.001]	
Educational attainment: middle	−0.011	0.007	−1.58	.115	[−0.025, 0.003]	
Educational attainment: high	−0.031	0.007	−4.47	**<.001** ***	[−0.045, −0.018]	
Income level: middle	0.000	0.007	0.01	.994	[−0.014, 0.014]	
Income level: high	−0.007	0.007	−0.98	.328	[−0.022, 0.007]	
Country of birth (−NL + OTH)	0.040	0.020	2.02	**.044** *	[0.001, 0.078]	
IDN (*n* = 3,855)						0.068
Effect (intercept)	2.746	0.003	893.42	**<.001** ***	[2.740, 2.752]	
Number of languages	−0.006	0.001	−4.29	**<.001** ***	[−.009, −0.003]	
L2 AoA	−0.004	0.001	−3.38	**<.001** ***	[−0.007, −0.002]	
Age	0.019	0.001	14.19	**<.001** ***	[0.016, 0.021]	
Sex (−F + M)	−0.007	3	−2.58	**<.01** **	[−0.012, −0.002]	
Educational attainment: middle	−0.003	0.003	−0.99	.320	[−0.010, 0.003]	
Educational attainment: high	−0.012	0.003	−3.39	**<.001** ***	[−0.018, −0.005]	
Income level: middle	−0.007	0.003	−1.97	**.048** *	[−0.013, −0.000]	
Income level: high	−0.011	0.004	−3.02	**<.01** **	[−0.018, −0.004]	
Country of birth (−NL+OTH)	−0.011	0.010	−1.11	.267	[−0.030, 0.008]	
OBK (*n* = 3,878)						0.046
Effect (Intercept)	1.246	0.007	173.21	**<.001** ***	[1.232, 1.260]	
Number of languages	0.024	0.003	7.21	**<.001** ***	[0.017, 0.030]	
Age	−0.016	0.003	−5.08	**<.001** ***	[−0.022, −0.010]	
Sex (−F + M)	−0.009	0.006	−1.40	.163	[−0.021, 0.004]	
Educational attainment: middle	0.009	0.008	1.10	.273	[−0.007, 0.024]	
Educational attainment: high	0.041	0.008	5.18	**<.001** ***	[0.026, 0.057]	
Income level: middle	0.020	0.008	2.57	**.010** *	[0.005, 0.036]	
Income level: high	0.029	0.009	3.41	**<.001** ***	[0.012, 0.050]	
Country of birth (−NL+OTH)	−0.013	0.023	−0.60	.547	[−0.059, 0.031]	
OCL (*n* = 3,887)						0.052
Effect (intercept)	0.918	0.004	223.58	**<.001** ***	[0.910, 0.926]	
Number of languages	0.013	0.002	7.09	**<.001** ***	[0.010, 0.017]	
Age	−0.008	0.002	−4.29	**<.001** ***	[−0.011, −0.004]	
Sex (−F + M)	−0.011	0.004	−3.27	**<.01** **	[−0.019, −0.005]	
Educational attainment: middle	0.021	0.005	4.53	**<.001** ***	[0.012, 0.030]	
Educational attainment: high	0.036	0.005	7.77	**<.001** ***	[0.027, 0.045]	
Income level: middle	0.008	0.005	1.67	.095	[−0.001, 0.016]	
Income level: high	0.007	0.005	1.41	.158	[−0.003, 0.016]	
Country of birth (−NL+OTH)	0.011	0.013	0.82	.410	[−0.015, 0.036]	

*Notes:* CI = confidence interval; DET = Detection task (processing speed); Est. = estimate; F = female; IDN = Identification task (attention); M = male; NL = the Netherlands; OBK = One Back task (working memory); OCL = One Card Learning task (recognition memory); OTH = other country; SE = standard error. Reference level Education attainment = low, reference level Income level = low. 95% confidence intervals (CIs) represent the bootstrapped confidence intervals. Values in bold reflect significance at least the *p* < .05 level.

* *p* < .05.

** *p < *.01.

*** *p* < .001.

**Table 4.** Effect Sizes (Semi-Partial *R*^2^) of the Predictors in the Linear Regression Models

Variable	DET	IDN	OBK	OCL
Number of languages	0.008	0.004	0.013	0.012
L2 age of onset of acquisition	—	0.003	—	—
Age	0.041	0.049	0.006	0.004
Sex	0.001	0.002	0.000	0.003
Educational attainment	0.005	0.003	0.007	0.015
Income level	0.000	0.002	0.003	0.000
Country of birth	0.000	0.000	0.000	0.000

*Notes:* DET = Detection task (processing speed); IDN = Identification task (attention); OBK = One Back task (working memory); OCL = One Card Learning task (recognition memory).

#### Detection task

For the Detection task, the predictor “number of languages learned” improved the fit of the demographics model (𝜒^2^(1) = 27.68, *p* < .001). A higher number of languages learned was related to faster RTs on the Detection task measuring processing speed (*p* < .001, *sr*^2^ = 0.008). None of the other multilingual experience variables (L2 proficiency, L2 AoA, frequency of multilingual language use, and language switching habits) contributed to the model fit for the Detection task.

#### Identification task

The addition of the number of languages learned to the demographics model also improved the model fit for the Identification task (𝜒^2^(1) = 16.88, *p* < .001). Having learned more languages throughout life was associated with faster RTs on the Identification task assessing attention (*p* < .001, *sr*^2^ = 0.005). In addition, L2 AoA significantly contributed to this model (𝜒^2^(1) = 4.58, *p* < .05). A later L2 AoA was associated with faster RTs (*p* < .001, *sr*^2^ = 0.003). The model fit for the Identification task did not improve by adding the other multilingual experience variables.

#### One Back task

For the One Back task, only the number of languages predictor improved the demographics model fit (𝜒^2^(1) = 37.43, *p* < .001). A higher number of learned languages was related to a higher hit rate on this task assessing working memory (*p* < .001, *sr*^2^ = 0.013).

#### One Card Learning task

Finally, for the One Card Learning task, the number of languages also contributed to the model fit (𝜒^2^(1) = 42.52, *p* < .001). A higher number of learned languages was associated with a higher hit rate on this task, such that the more languages were learned in life, the higher the hit rate on the One Card Learning task assessing recognition memory (*p* < .001, *sr*^2^ = 0.012). The model fit did not improve by adding the other multilingual experience variables.

We also modeled the interaction between educational attainment and the number of languages learned for the four separate Cogstate tasks to assess the possibility that individuals with lower educational attainment show greater effects of having learned more languages in life. We did not observe any significant interactions. This suggests that having learned more languages is associated with better cognitive functioning in our older adult sample regardless of the educational level obtained.

In sum, the number of languages learned predicted the performance on all four Cogstate subtasks, but its effect size was consistently small (*sr*^2^ ≤ 0.015) in all analyses, especially compared with the effect sizes of age at assessment for the Detection and Identification tasks, which are based on reaction time (see Table 4). At the same time, the effect sizes of the number of languages learned and educational attainment were comparable. Apart from L2 AoA for the Identification task, the effects of other multilingual experience variables did not reach significance.

## Discussion

This study investigated the relationship between knowledge-based and usage-based multilingual language experience variables and cognitive functioning in a large cohort of older adults in the diversely multilingual northern Netherlands. To this end, processing speed, attention, working memory, and recognition memory, measured by the Cogstate Brief Battery, were related to several multilingual experience variables in a sample of 3,972 participants aged 55 years or older in the Lifelines Cohort Study. These variables included the knowledge-based variables of the number of languages learned, L2 proficiency, and L2 AoA, as well as the usage-based variables of frequency of multilingual language use and language switching. Our results show that the number of languages someone had learned during their lifetime was significantly and positively associated with better attention, processing speed, working memory, and recognition memory. Additionally, a later L2 AoA was associated with better attention. The effects of these knowledge-based multilingual experiences reached significance even when controlling for demographic variables (i.e., age, education level, net income level, and country of birth), but the effect sizes were consistently small. Together, this suggests that there may be a robust, but small accumulative beneficial effect of the number of languages learned on cognitive functioning in older adulthood.

A strength of our study was that it treated multilingualism as an experience, rather than as a unidimensional construct, by examining knowledge-based as well as usage-based variables. In addition, we contributed to the limited literature on the cognitive aging effects in multilinguals by investigating a sample comprising both bilinguals and multilinguals. Previous research on bilingual individuals has documented positive associations between bilingualism and cognitive functioning, for example in processing speed (Grant and Dennis, 2017) and working memory (Grundy & Timmer, 2017). Our study extends these findings by showing that each additional language learned on top of the two already known adds a small but statistically significant benefit to cognitive functioning in these cognitive domains. Our findings provide new insights into the relationship between multilingualism and cognition in older adults in the northern Netherlands, suggesting that knowing more than two languages may offer incremental cognitive performance compared with bilingualism alone. This aligns with Ihle et al. (2016) and Kavé et al. (2008), who also found additive effects of the number of known languages on cognitive functioning in older adults. Interestingly, like in our study, the authors concluded this on the basis of a large population-based sample.

Our large sample size is, especially in comparison to previous work in this field (Van den Noort et al., 2019), another merit of our study. Large participant samples allow for the detection of relatively small effects. Arguably, previous work with smaller sample sizes has therefore not always been able to detect a significant effect of multilingual experience on cognition. Potentially, multilingual experience effects are “overshadowed” by other, considerably large effects on cognitive functioning in these cases, such as age. We note that, in our study, the number of languages learned yielded comparable effect sizes to educational attainment, and the number of languages learned even explained more variance in the older adults’ working memory performance. Thus, despite having a small effect, the number of languages learned in life may be a meaningful predictor of cognitive function in later life and we recommend that its significance should be considered alongside well-known cognitive reserve proxies, such as educational attainment, in future work as well. It must be acknowledged that not all research groups have access to such large multilingual samples as examined in our study. Possible and interesting avenues for investigating the effects of multilingual experiences on aging in smaller multilingual samples could be done through dense repeated measurements of cognition to increase power (e.g., Kliesch et al., 2021) or to supplement a quantitative approach with exploratory, qualitative research to detail to what extent older adults feel their multilingual experience has been instrumental in their quality of life with respect to cognitive as well as other domains of aging. Despite sample size often being a key factor in detecting effects, we would like to note that differences in sample size alone cannot fully explain the field’s mixed findings. Other factors related to methodology, such as the choice of cognitive measurement, are likely to also play a role (see below).

In addition to a higher number of languages learned, our analysis revealed that a later L2 AoA was also associated with better attention, also with a small effect size. This may initially seem counterintuitive, considering how more years of “training” in multilingual language regulation supposedly leads to enhanced cognitive performance. Schroeder and Marian (2017) offer a compelling possible explanation for this finding, however, arguing on the basis of the demand-supply mismatch theory (e.g., Lövdén et al., 2010) that learning and speaking more than one language may lead to cognitive adaptations only in some instances. The general idea is that an increase in supply (i.e., cognitive abilities) occurs when someone’s current supply is challenged by the demand of the environment, resulting in cognitive adaptations to meet this demand. This may especially be the case for multilinguals who learn their languages sequentially, rather than simultaneously, considering that the increase in demand occurs more gradually in sequential multilinguals. According to this reasoning, sequential multilinguals should build up more cognitive reserve than simultaneous multilinguals, resulting in superior cognitive performance in later life. In the north of the Netherlands, many (older adult) L1 speakers of a regional language such as Frisian or Low Saxon will have only started learning the majority language, Dutch, when entering school (typically at age 6). Additionally, inhabitants of this region typically have the opportunity to speak other languages, such as English and German, which are most commonly (formally) learned in secondary education. The languages that are learned at a later age therefore require different, and perhaps more cognitively taxing, strategies to learn and use than languages that are learned more automatically and implicitly, for instance from birth. As a result, cognitive functions may be differentially affected in those multilinguals who acquire languages after the initial language acquisition phase.

Our results suggest that this effect of later L2 AoA may not hold for cognitive functions other than attention. It is not fully clear why L2 AoA emerged as a significant predictor for only the attention component of cognitive functioning in our study. Attention has been proposed as a mechanism especially relevant for learning an L2 in the classroom and subsequently using it (Robinson et al., 2012); arguably, it is then especially attention that is trained as a result of sequential L2 acquisition and is consequently better preserved in older age. Importantly, our finding also has interesting implications for investigating language learning in older adulthood as a cognitive training aiming to enhance cognitive reserve (Antoniou et al., 2013), especially in (functionally) monolingual older adults whose cognitive abilities are taxed to meet the sudden demand of learning and using another language in addition to their first one.

Besides the small effects of the knowledge-based multilingual experience variables of the number of languages learned and L2 AoA, the usage-based experiences examined in our study did not significantly predict cognitive functioning in our analyses. Referring back to the sample size discussion above, intricate individual differences related to the more dynamic aspects of multilingual experience may not have been associated with significant cognitive effects in our sample presumably because these effects related to language use are either too small, simply non-existent, and/or do not contribute to cognition in addition to other multilingual experience variables or demographic characteristics. Although our results replicate previous work, our findings do not match Pot et al.’s (2018) in this respect, who found that not merely the knowledge of multiple languages, but rather the amount of L2 use in combination with openness to new experience was associated with cognitive functioning in older adults in the northern Netherlands. In addition to a difference in sample size, this contrast in results potentially emerged because of the difference in enquiring about language use between Pot et al.’s study and ours. Our study asked about the frequency of using multiple languages (ranging from daily to once a month), and not about the amount of L2 use relative to L1 use in certain contexts, for instance (Green and Abutalebi, 2013). Alternatively, the discrepancy could originate from the difference in cognitive measurement. Whereas Pot et al. found an effect on cognitive functioning as measured using the Flanker task, tapping inhibitory control, our study examined processing speed, attention, working memory, and recognition memory using the Cogstate Brief Battery. As such, it is possible that multilingual language use by older adults in the northern Netherlands is specifically associated with executive functions such as inhibitory control, but not necessarily with other cognitive functions (cf. Calvo et al., 2016). Future work would do well to replicate this work using comparable methodology in different multilingual populations to shed additional light on the complex interaction between knowledge-based and usage-based multilingual experiences and cognitive aging.

Despite our study’s merits, we also have to acknowledge a few limitations. As our study was based on retrospective data, we cannot infer any causal effects of multilingual experience factors on cognitive functioning. Older adulthood is characterized by an accumulation of life experiences and, as such, the direct effects of multilingualism on cognition are difficult to disentangle from these other experiences, especially with regard to usage-based multilingual experience variables that tend to vary across the lifespan. Although we controlled for demographic variables in our models that are known to confound with multilingualism and cognition, it cannot be ruled out that an unknown explanatory factor is confounded with the number of languages learned. Furthermore, participants with higher cognitive capacity are potentially more likely to learn more languages throughout life. However, many people in the north of the Netherlands are bilingual or multilingual from a young age, especially the generations included in our sample. As such, speaking multiple languages may not necessarily be due to a talent or interest in learning languages, but is rather a consequence of the environment (Bialystok et al., 2012). Furthermore, the design of the current study only allows inferences about the older adults’ cognitive *state* and not about multilingual experiences mitigating cognitive *decline* or increasing *reserve.* Therefore, a longitudinal evaluation of the relationship between multilingual experiences and cognitive functioning from mid-adulthood to older adulthood is needed. Ideally, this would include an investigation of multilinguals presenting with AD pathology or having been diagnosed with MCI or a type of dementia, considering that these are situations in which individual differences in cognitive reserve are of greatest importance.

In conclusion, this study has shown that the knowledge-based experiences of a higher number of languages learned and a later age of L2 acquisition relate to better cognitive functioning in multilingual older adults in the northern Netherlands. Although a longitudinal evaluation of cognitive functioning in relation to multilingual experience is warranted to provide stronger evidence for the hypothesis that learning and/or using multiple languages fosters healthy cognitive aging, our study’s results suggest that learning more languages throughout life may promote better cognition in older adulthood. This study zoomed in specifically on multilingualism as a potential cognitive reserve contributor; for one, because multilingualism is a complex, sustained experience, but also because it can potentially have broader effects on aging apart from merely cognitive reserve and/or state. For example, the multitude of social networks related to being able to speak multiple languages could potentially contribute to a higher quality of life in older adulthood. Despite these potential implications for healthy aging, multilingualism has been merely glanced over in medical and neuroscientific research more broadly (Voits et al., 2022). We hope that future studies enquiring about healthy cognitive aging will involve interdisciplinary investigations that consider multilingual experience as a potential cognitive reserve contributor.

## Supplementary Material

gbae200_suppl_Supplementary_Tables_S1-S3

## Data Availability

The data catalog of Lifelines Cohort Study is publicly accessible on https://data-catalogue.lifelines.nl. These data may be obtained from a third party and are not publicly available. Researchers can apply for data used in this study at the Lifelines research office (research@lifelines.nl). More information about how to request Lifelines data and the conditions of use can be found on their website (https://www.lifelines-biobank.com/researchers/working-with-us/step-1-prepare-and-submit-your-application). The scripts used to preprocess and analyze the data are available at https://osf.io/3465d/?view_only=b4e9efcf22f3422694d3837ef32aaa4c. The study was not pre-registered.

## Author Note

1. To better describe our study sample, this paper adopts “multilingual experience” as an inclusive umbrella term for both bilingualism (generally, the use of two languages) and multilingualism (sometimes defined as the use of three or more languages), following Cenoz (2013). By doing so, however, we do not intend to rule out the possibility that bilingualism and multilingualism may have distinct neurocognitive correlates.
