# Supplementary material for: The Association Between Multilingual Experience Factors and Cognitive Functioning in Older Adults: A Lifelines Study
Source: J Gerontol B Psychol Sci Soc Sci. 2025 Jan 11;80(3):gbae200. doi: 10.1093/geronb/gbae200 (PMC11815170; doi:10.1093/geronb/gbae200)
Supplement: gbae200_suppl_Supplementary_Tables_S1-S3 [file gbae200_suppl_supplementary_tables_s1-s3.docx]

***The Journals of Gerontology, Series B: Psychological Sciences and Social Sciences* Supplementary Material: van den Berg et al. The association between multilingual experience factors and cognitive functioning in older adults: A Lifelines study.**

### **Multilingual experience questionnaire**

*Which languages have you learned? We also consider dialects to be separate languages. When we refer to languages in the remainder of the questionnaire, we automatically mean dialects as well.*

**LANG01A: Which languages have you learned? [the questions LANG01A–LANG01E were repeated for up to six languages]**

**Please start with the language that you consider to be your mother tongue or first language.**

1 = Dutch

2 = "Drenths" dialect [dialect of Low Saxon]

3 = German

4 = English

5 = French

6 = Frisian [minority language spoken in the province of Fryslân]

7 = "Gronings" dialect [dialect of Low Saxon]

8 = Italian

9 = Spanish

10 = Other, namely…

*Please indicate here how well you are able to understand and speak this language. 10 is the highest mark and 1 is the lowest, but 10 does not necessarily imply perfect mastery. Please use a 10 for your first language as a reference point.*

**LANG01B: How well can you understand the language when you hear it being spoken?**

[1 - 10]

**LANG01C:** **How well are you able to speak this language?**

[1 - 10]

**LANG01D: How old were you when you first started learning this language (in years)? If you learned the language from birth, please fill in 0 here.**

**LANG01E: Did you learn this language in a school setting (at school or through a course) or outside of a formal instruction context?**

1 = In a school setting

2 = Outside of a formal instruction context

3 = Both

**LANG11: Would you refer to yourself as multilingual?**

1 = Yes

2 = No

**LANG11A: Do you view your own multilingualism as something positive?**

1 = Yes

2 = No

**LANG11ATXT: Could you briefly explain why you indicated {yes/no} in response to the previous question?**

**LANG12: Do you currently still use multiple languages in your daily life?**

1 = Yes

2 = No

**LANG12A [if LANG12 = No]: At what age did you stop using multiple languages?**

**LANG13: Do you often switch between the languages that you speak?**

1 = No, I use my languages in different settings and situations (I use one language at home, for instance, and another language outside of the home or I use one language with certain people and the other with other people).

2 = Yes, I sometimes switch languages within one situation or setting.

3 = Yes, I frequently switch languages within one situation or setting.

**LANG14A: With whom do you use multiple languages [multiple answers possible]?**

1 = Relatives

2 = Friends

3 = Nuclear family members

4 = Colleagues

5 = Professional partners and business partners

6 = Other, namely…

**LANG14B: In which situations or settings do you use multiple languages [multiple answers possible]?**

1 = At home

2 = At work

3 = When I am at a club (social or sports club)

4 = For professional purposes

5 = Other, namely…

**LANG15: Have you ever been in a situation where you did hear another language being spoken around you but did not speak this language yourself? (Here, we mean your everyday living situation and not when on holiday)**

1 = Yes

2 = No

**LANG15TXT [if LANG15 = Yes]: Please indicate here when you found yourself in such a situation and which languages were involved.**

**LANG16: How often do you currently use multiple languages or have you used multiple languages in the past?**

1 = Every day

2 = More than once a week

3 = Once a week

4 = Once a month

5 = Less than once a month

**LANG17: Would you like to share something else in relation to your language use and multilingualism? We would love to hear that.**

### **Description of Cogstate subtasks**

***Detection task (processing speed)***

The Detection task served to measure psychomotor functioning and processing speed. In this task, participants were instructed to press a key on the keyboard corresponding to “Yes” as soon as the playing card in the center of the screen turned face up. The task ended after the participant had completed 35 correct trials. The primary outcome for the Detection task was reaction time in milliseconds for correct responses, where a faster reaction time indicates a better performance.

***Identification task (attention)***

In the Identification task, which measured visual attention, participants were asked to press a key corresponding to “Yes” if the playing card in the center of the screen was red, and a key corresponding to “No” if it was not. In order for the task to end, 30 correct responses were required. Reaction time in milliseconds for correct responses was the main outcome, where a faster reaction time indicates a better performance.

***One Back task (working memory and attention)***

Working memory and attention was assessed by means of the One Back task, in which participants were instructed to press the “Yes” key if the playing card in the center of the screen was the same as that on the previous trial, and the “No” key if this was not the case. The task ended after 30 correct trials. The outcome measure that was used in the analyses was the proportion of correct answers (i.e., hit rate), where a higher hit rate indicates better performance.

***One Card Learning task (recognition memory)***

The final task, the One Card Learning task, measured visual learning and recognition memory. Six cards were randomly drawn from the deck and reappeared throughout the task, alternating with non-repeating cards. Participants were required to press the “Yes” key if they had seen the playing card in the center of the screen before in the task, and press “No” if this was not the case. Participants had to complete 42 trials before the task ended; unlike for the other tasks, there was no criterion for the number of correct answers. The proportion of correct answers (i.e., hit rate) was the main outcome resulting from this task. A higher hit rate indicates better performance.

###

### **Data preparation and model building**

Data were preprocessed and analyzed using R (version 4.2.0, R Core Team, 2022). The dataset was tidied and cleaned using the *dplyr* package (Wickham et al., 2018).

The primary outcomes from each Cogstate subtest were subjected to linear regression analysis and were entered as dependent numerical variables. First, a demographics model was built including age, gender, educational level, income level, and country of birth. Categorical variables with two levels (i.e., gender and country of birth) were coded with sum-to-zero orthogonal contrasts to improve the interpretation of the results (Baguley, 2012; Schad et al., 2020). For gender, ‘female’ was coded as -0.5 and ‘male’ was coded as +0.5 (-F+M). For country of birth, we coded ‘the Netherlands’ as -0.5 and ‘other country’ as +0.5 (-NL+OTH). The effects of the variables gender and country of birth reflect the change in effect when going from one level to the other.

We built step-wise models and used the Akaike information criterion (AIC; Cavanaugh & Neath, 2019) to assess the contribution of each language variable to the demographics model fit, and, as such, to examine the predictive value of individual multilingual experience variables to cognitive functioning. Comparing the AIC between models determines whether enough additional variability is explained in the dependent variable to justify the additional complexity in the model. In other words, if the AIC difference between a simpler and a more complex model is not significant, adding a predictor to the simpler model is not justified. As such, the predictors that did not significantly contribute to a better regression model fit were omitted from subsequent models in our analyses. Demographic variables expected to confound with cognition and multilingual experience, although not significant, were kept in the final model as these were part of the base mode controlling for these potential effects.

T-tests using Satterthwaite approximations to degrees of freedom from the *lmerTest* package (Kuznetsova et al., 2017) were used to obtain the *p*-values of the estimates. All numerical predictors in the models were *z*-scaled and centered around the mean. We found no evidence of multicollinearity between the language and demographic variables that were included in the models. Model criticism was applied to the best-fitting models by excluding all observations with absolute residuals larger than 2.5 SDs above the mean. The model summaries in the article reflect the results of these trimmed datasets. 95% confidence intervals were obtained using bootstrap analysis with 100 iterations using the *boot* package (Canty & Ripley, 2021; Davison & Hinkley, 1997). Effect sizes (semi-partial R-squared [*sr*^2^]) of the model predictors were calculated using the *effectsize* package (Ben-Shachar et al., 2020). *Sr*^2^ estimates the contribution of each model term to the total explained variance (*R*^2^) of the regression model.

**Supplementary Table 1.** The most commonly reported spoken languages in the sample.

|  | **Learned languages** |
| --- | --- |
| L1  (*n* = 3,972) | Dutch: 55%  Frisian: 23%  Low Saxon: 19%  Other dialect: 1%  English: 0.5%  German: 0.5%  French: < 10  Indonesian/Malay: < 10  Other language: < 10  Scandinavian language: <10  Slavic language: < 10  Surinamese/Caribbean: < 10 |
| L2  (*n* = 3,972) | Dutch: 41%  English: 24%  Low Saxon: 13%  German: 11%  Frisian: 8%  French: 2%  Other dialect: 1%  Indonesian/Malay: < 10  Other language: < 10  Surinamese/Caribbean: < 10 |
| L3 (*n* = 3,543) | English: 48%  German: 37%  French: 7%  Frisian: 3%  Low Saxon: 3%  Dutch: 1% |
| L4 (*n* = 2,947) | German: 37%  French: 30%  English: 25%  Low Saxon: 3%  Frisian: 2%  Dutch: 1%  Other language: 1% |
| L5 (*n* = 2,090) | French: 52%  Low Saxon: 12%  German: 10%  Frisian: 9%  Other language: 7%  English: 6%  Classical language: 1%  Dutch: 1%  Scandinavian language: 1%  Other dialect: 1% |
| L6 (*n* = 712) | Low Saxon: 28%  Other language: 19%  Frisian: 17%  French: 12%  Classical language: 7%  Slavic language: 4%  German: 3%  Other dialect: 3%  MENA language: 2%  English: 2%  Scandinavian language: 2%  Indonesian/Malay: < 10 |

*Note.* Percentages are rounded. To avoid identifying individuals, in case of an *n* of < 10 for a specific response category, the percentage is not mentioned for that response category and the closest-consecutive response category.

**Supplementary Table 2.** Additional descriptives of multilingual experience in terms of age of onset of acquisition, proficiency, and learning context of the learned languages (M, SD, or percentages), stratified by the number of languages learned by participants.

|  | **Two**  **(*n* = 430)** | **Three**  **(*n* = 595)** | **Four**  **(*n* = 857)** | **Five**  **(*n* = 1,378)** | **Six**  **(*n* = 495)** | **Seven or more  (*n* = 217)** |
| --- | --- | --- | --- | --- | --- | --- |
| L1 AoA | 0.80 (2.10) | 0.72 (2.27) | 0.54 (1.82) | 0.35 (1.27) | 0.58 (1.69) | 0.46 (1.43) |
| L1 LearnContext % in school context  % outside school context % both | 14% 39% 46% | 13% 35% 52% | 11% 28% 61% | 8% 34% 57% | 7% 34% 59% | 8% 28% 65% |
| L2 AoA | 7.63 (8.68) | 8.07 (6.65) | 8.99 (5.66) | 6.67 (5.42) | 6.38 (5.55) | 7.37 (6.09) |
| L2 ProfSpeak (1-10) | 8.04 (1.86) | 8.15 (1.73) | 7.83 (1.77) | 8.53 (1.68) | 8.54 (1.73) | 8.24 (1.67) |
| L2 ProfUnd (1-10) | 8.41 (1.73) | 8.57 (1.51) | 8.27 (1.61) | 8.99 (1.32) | 9.04 (1.29) | 8.78 (1.39) |
| L2 LearnContext % in school context  % outside school context % both | 45% 21% 34% | 42% 17% 41% | 46% 15% 40% | 34% 23% 44% | 31% 28% 41% | 26% 24% 50% |
| L3 AoA | - | 15.29 (9.95) | 13.12 (5.07) | 12.37 (3.44) | 12.17 (4.56) | 11.92 (6.77) |
| L3 ProfSpeak (1-10) | - | 5.53 (1.92) | 6.07 (1.65) | 6.3 (1.61) | 6.75 (1.71) | 6.81 (1.75) |
| L3 ProfUnd (1-10) | - | 6.24 (1.87) | 6.77 (1.63) | 6.97 (1.57) | 7.54 (1.55) | 7.78 (1.53) |
| L3 LearnContext % in school context  % outside school context % both | - | 60% 18% 22% | 62% 10% 29% | 71% 4% 25% | 59% 11% 30% | 51% 13% 36% |
| L4 AoA | - | - | 14.47 (8.53) | 12.95 (5.54) | 12.57 (3.16) | 12.20 (4.96) |
| L4 ProfSpeak (1-10) | - | - | 5.11 (1.97) | 5.44 (1.83) | 5.87 (1.79) | 5.90 (1.91) |
| L4 ProfUnd (1-10) | - | - | 5.85 (2.09) | 6.19 (1.89) | 6.63 (1.80) | 6.91 (1.86) |
| L4 LearnContext % in school context  % outside school context % both | - | - | 63% 16% 21% | 77% 4% 19% | 72% 6% 22% | 65% 6% 29% |
| L5 AoA | - | - | - | 15.24 (11.59) | 15.37 (10.77) | 16.99 (12.90) |
| L5 ProfSpeak (1-10) | - | - | - | 4.38 (2.38) | 4.83 (2.26) | 5.16 (2.12) |
| L5 ProfUnd (1-10) | - | - | - | 5.21 (2.55) | 5.80 (2.36) | 6.43 (2.20) |
| L5 LearnContext % in school context  % outside school context % both | - | - | - | 67% 22% 11% | 67% 19% 14% | 55% 22% 23% |
| L6 AoA | - | - | - | - | 24.96 (17.71) | 21.72 (14.76) |
| L6 ProfSpeak (1-10) | - | - | - | - | 4.65 (2.49) | 4.51 (2.30) |
| L6 ProfUnd (1-10) | - | - | - | - | 6.28 (2.72) | 6.34 (2.51) |
| L6 LearnContext % in school context  % outside school context % both | - | - | - | - | 38% 51% 11% | 38% 47% 14% |

**Supplementary Table 3.** Correlation matrix of the variables included in the linear regression analyses, including continuous (Spearman’s rho), dichotomous, polychoric, and polyserial correlations.

|  | **Age** | **NumbLang** | **L2 Profspeak** | **L2 AoA** | **MultiUseFreq** | **SwitchFreq** | **EduLevel** | **Income** | **Gender** | **CountryofBirth** |
| --- | --- | --- | --- | --- | --- | --- | --- | --- | --- | --- |
| **Age** | **1** | .02 | -.01 | .03 | .02 | .00 | .03 | .03 | .15 | .00 |
| **NumbLang** | .02 | **1** | .10 | -.08 | -.08 | .06 | .40 | -.15 | -.01 | .01 |
| **L2 ProfSpeak** | -.01 | .10 | **1** | -.48 | -.33 | .24 | -.03 | .01 | -.05 | -.07 |
| **L2 AoA** | .03 | -.08 | -.48 | **1** | .24 | -.14 | .09 | -.02 | .01 | .21 |
| **MultiUseFreq** | .02 | -.08 | -.33 | .24 | **1** | -.45 | .01 | .00 | -.02 | .04 |
| **SwitchFreq** | .00 | .06 | .24 | -.14 | -.45 | **1** | .05 | -.07 | .02 | .01 |
| **EduLevel** | .03 | .40 | -.03 | .09 | .01 | .05 | **1** | -.28 | .24 | .16 |
| **Income** | .03 | -.15 | .01 | -.02 | .00 | -.07 | -.28 | **1** | -.07 | -.05 |
| **Gender** | .15 | -.01 | -.05 | .01 | -.02 | -.02 | .24 | -.07 | **1** | .05 |
| **CountryofBirth** | .00 | .01 | -.07 | .21 | .04 | -.01 | .16 | -.05 | .05 | **1** |

**References**

Baguley, T. (2012). *Serious stats: A guide to advanced statistics for the behavioral sciences*. Macmillan International Higher Education.

Ben-Shachar, M. S., Lüdecke, D., & Makowski, D. (2020). effectsize: Estimation of Effect Size Indices and Standardized Parameters. *Journal of Open Source Software,* *5*(56), 2815. https://doi.org/10.21105/joss.02815

Canty, A., & Ripley, B. D. (2021). *boot: Bootstrap R (S-Plus) Functions*.

Cavanaugh, J. E., & Neath, A. A. (2019). The Akaike Information Criterion: Background, derivation, properties, application, interpretation, and refinements. *WIREs Computational Statistics*, *11*(3), e1460. https://doi.org/10.1002/wics.1460

Davison, A. C., & Hinkley, D. V. (1997). *Bootstrap methods and their applications*. Cambridge University Press. http://statwww.epfl.ch/davison/BMA/

Kuznetsova, A., Brockhoff, P. B., & Christensen, R. H. B. (2017). lmerTest Package: Tests in Linear Mixed Effects Models. *Journal of Statistical Software, 82*, 1–26. https://doi.org/10.18637/jss.v082.i13

Schad, D. J., Vasishth, S., Hohenstein, S., & Kliegl, R. (2020). How to capitalize on a priori contrasts in linear (mixed) models: A tutorial. *Journal of Memory and Language*, 110, 104038.

Wickham, H., François, R., Henry, L., & Müller, K. (2018). *dplyr: A Grammar of Data Manipulation*. https://CRAN.R-project.org/package=dplyr
